# Imbalance of ω3 and ω6 Fatty Acids in Breast Milk of Overweight/Obese Women

**DOI:** 10.3390/nu17132158

**Published:** 2025-06-28

**Authors:** Michael G. Ross, Manasa P. Kavasery, Guang Han, MacKenzie K. Cervantes, Lihiri Bora, Kevin J. Williams, Mina Desai

**Affiliations:** 1 The Lundquist Institute at Harbor-UCLA Medical Center, Torrance, CA 90502, USA; mkavasery@dhs.lacounty.gov (M.P.K.); ghan@lundquist.org (G.H.); mkerr@lundquist.org (M.K.C.); lihiri.bora@lundquist.org (L.B.); mdesai@lundquist.org (M.D.); 2 Department of Obstetrics and Gynecology, David Geffen School of Medicine, University of California, Harbor-UCLA Medical Center, Torrance, CA 90502, USA; 3 Department of Obstetrics and Gynecology, Charles R. Drew University, Los Angeles, CA 90059, USA; 4 Department of Biological Chemistry, David Geffen School of Medicine, University of California, Los Angeles, CA 90095, USA; lipidomics@mednet.ucla.edu

**Keywords:** foremilk and hindmilk breast milk, maternal body mass index, polyunsaturated fatty acids, linoleic acid, α-linolenic acid, arachidonic acid, docosahexaenoic acid

## Abstract

**Background/Objectives**: Studies demonstrate better health outcomes for infants consuming milk with higher concentrations of ω3 (ALA and DHA) and negative health outcomes associated with higher ω6 (LA and AA) PUFAs. We studied the relationship between maternal BMI and PUFA levels in maternal plasma and breast milk. **Methods**: Women at 7–8 weeks postpartum were grouped according to normal BMI (18–24.9 kg/m^2^) and overweight/obese (OW/OB; ≥25 kg/m^2^). Maternal blood and continuous breast milk samples obtained from foremilk to hindmilk were analyzed for lipidomics. **Results**: The plasma levels of ω3 and ω6 PUFA were significantly lower in OW/OB subjects, with a total ω3 and ω6 FA level of 50% for women with normal BMI. Conversely, breastmilk levels of total ω3 and ω6, including their respective precursors of LCFAs (ALA and LA), were significantly increased in both foremilk and hindmilk samples of OW/OB. Despite this, DHA (ω3 PUFA) levels in OW/OB women were similar in foremilk and significantly decreased in hindmilk samples as compared to normal BMI women. Consequently, the ratio of DHA/Total ω3 significantly decreased in foremilk and hindmilk samples of OW/OB women. However, proinflammatory AA (ω6 PUFA) levels increased, resulting in an increased ratio of AA/DHA in OW/OB women. Breast milk DHA was positively correlated, whereas AA was negatively correlated with maternal plasma. **Conclusions**: Marked differences in maternal plasma and breast milk ω3 and ω6 FA concentrations among women with OW/OB indicate significant differences in nutritional exposures for their infants. Reduced milk DHA may be a consequence of reduced mammary peroxisomal conversion of ALA to DHA due to increased insulin/reactive species within the maternal obese environment. The imbalance of ω3 and ω6 FAs suggests that DHA supplementation and approaches to limit plasma to breast milk AA transfer in OW/OB subjects may be of value.

## 1. Introduction

Complementing the interaction of genes and the intrauterine environment on offspring phenotype [1], nutritional and environmental challenges during the newborn period further impact offspring plasticity. The profound and lasting impact of lactational programming, as evident in human and rodent studies, highlights the importance of breast milk composition, breastfeeding practices, and maternal health on an infant’s well-being and future health outcomes [2,3,4,5].

The World Health Organization (WHO) and the American Academy of Pediatrics recommend exclusive human milk feeding as the “normative standards for infant feeding” for the first six months of life [6,7] as breastfeeding infant health benefits include reduced infection, improved cognitive performance, and potentially reduced allergies and adult obesity [8]. Approximately 80% of women initiate breastfeeding at birth, with 60% continuing some breastfeeding at 6 months [9,10,11]. The lactational period, with variation in milk quantity and quality, feeding durations, maternal interactions, infant stress, and others, has a tremendous influence on newborn development with long-term health outcomes. The importance and evidence of lactational programming are demonstrated in rodent studies. Mice born to and nursed by obese dams develop early life and adult obesity [2,3]. However, the offspring of obese dams nursed by control dams demonstrate normal weight as adults, whereas the offspring of control dams nursed by obese dams develop adult obesity [2].

Human milk contains approximately 200 identified components, including solutions, colloids, membranes, globules, and live cells [12]. Typically, milk contains 0.8–0.9% protein, 3–5% fat, 6.9–7.2% carbohydrates, 0.2% minerals, and averages 66 kcal/100 mL [13,14,15,16] with an interquartile range of 62.0 to 72.5 kcal/100 mL, reflecting the significant individual variance. Milk synthesis is a complex process impacted by diet, serum composition, lipogenesis, and substrate uptake and synthesis by mammary epithelial cells. While the milk lipidome, proteome, metabolome, and small bioactives contribute to infant well-being, macronutrients are the primary determinants of milk caloric intake and infant growth. Milk carbohydrate is mainly lactose, while proteins comprise whey (α-lactalbumin, lactoferrin, β-lactoglobulin, albumin, and immunoglobulins) and casein (phosphoproteins) in addition to select hormones (e.g., leptin and ghrelin). Milk protein and carbohydrate content are remarkably stable.

Milk fats contribute to 40–50% of human milk calories [17], with triglycerides (TGs) accounting for 98% of milk lipid content. In contrast to relatively stable protein and carbohydrate content, milk fatty acid (FA) composition is highly variable, being influenced by maternal BMI and diet [15,16,18,19,20,21,22,23]. Milk fat is primarily contained within milk fat globules, a triglyceride-rich core surrounded by a tri-layer membrane. In mature human milk, TGs in the milk fat globule core consist mainly of long-chain FAs (LCFAs). Among these LCFAs, the essential FAs, which must be obtained via a maternal diet, comprise two families: Omega-6 (ω6) and Omega-3 (ω3). Specifically, the polyunsaturated FAs (PUFAs) 18:2n-6 linoleic (LA) [24] and 18:3n-3 α-linolenic acid (ALA) are precursors of longer chain C20 and C22 PUFAs [20], including arachidonic (ω6 AA), eicosapentaenoic (ω3 EPA), and docosahexaenoic acids (ω3 DHA). Importantly, half of all brain DHA accumulates during fetal and infant life [25] and ω3 FAs play a critical role in infant growth, neurodevelopment [26,27] and visual acuity in early life [28].

Maternal serum DHA deficiency is particularly marked in women with obesity, as obese pregnant women were three times as likely to be in the lowest tertile [29]. In regard to breast milk, a systematic review demonstrated that maternal BMI was associated with a lower milk ω3:ω6 ratio [30]. Thus, infants of overweight/obese (OW/OB) women may be provided an imbalance of ω3:ω6 FAs during this critical developmental period.

Our studies [31,32] and others [33,34] have demonstrated that human breast milk fat concentration increases dramatically (4-fold) from foremilk to hindmilk. Yet nearly all studies of human breast milk are limited to single milk samples, providing an incomplete assessment of the ω3 and ω6 content. In the present study, we specifically sought to examine the ω3 and ω6 PUFA content of human milk from foremilk to hindmilk in women with OW/OB and women with normal BMI. We further assessed the maternal serum concentrations of ω3 and ω6 FAs to examine the correlation with milk content and provide insight into potential metabolic mechanisms of breast milk FA production.

## 2. Materials and Methods

*Study Participants:* The study was approved by the Institutional Review Board at the Lundquist Institute at Harbor-UCLA Medical Center, Los Angeles, CA, USA. Women delivering singleton, term pregnancies were enrolled after written human subjects’ consent was obtained (Study # 32035-01). The exclusion criteria included women with breast implants, prior breast surgery, flat/inverted nipples, tongue-tie, low birth weight infants (<2500 g), or pregestational diabetes. All mothers were selected for Body Mass Index (BMI) based on pre-pregnancy reported weight and height, and were committed to exclusive breastfeeding for at least two months. Mothers were grouped according to normal BMI (18–24.9 kg/m^2^) and overweight/obese (OW/OB; ≥25 kg/m^2^), and studies were performed at 7–9 weeks postpartum (mature milk). Sample size (80% power, alpha 0.05) based upon a predicted mean fat content of 1.5 ± 0.2 and 1.8 ± 0.2 g/dL in normal BMI and OW/OB groups, respectively, required seven patients in each group. A total of 20 women were studied, of which 11 were OW/OB and 9 were of normal BMI.

Following informed consent, all women were interviewed by the staff for demographic information on ethnicity, maternal age, and pre-pregnancy height and weight. Additional medical data (height and weight at last prenatal visit, gestational age at delivery, pregnancy complications including gestational diabetes, medications, mode of delivery, and baby’s length and weight at birth and discharge, and gender) were obtained from the electronic medical record or patient interview. Studies were performed in the Lundquist/Harbor-UCLA Clinical and Translational Research Center (CTRC) outpatient clinic. All studies took place between 10 a.m. and 12 p.m. to avoid potential circadian changes in breast milk composition.

*Blood Collection and Breast Milk Sampling:* Mothers abstained from eating or drinking for more than one hour prior to the study, and had refrained from breastfeeding for at least more than one and a half hours from the prior infant breastfeeding, which was from only one breast. Maternal blood samples were collected before breast milk sampling, and the milk samples were collected from the breast opposite to that which was used for the prior feed (preceding the study). The nipple and areola were wiped clean, and an electrical pump (Medela, McHenry, IL, USA) was applied to the breast. Pumped continuous breast milk samples were obtained in 10 mL aliquots until the breast was emptied or there was no further milk production. Maternal plasma and milk samples were frozen at −80 °C in polypropylene tubes for fatty acid analysis.

Blood Analysis: Serum samples were sent to Quest Diagnostics (West Hills, CA, USA) for analysis of triglycerides and insulin (immunoassay).

*Fatty Acid Analysis:* This was undertaken at the UCLA Lipidomics Core. Centrifuged plasma (25 µL) and milk (25 µL) samples were extracted using a modified Bligh and Dyer extraction [35]. An internal standard mixture consisting of 70 lipid standards across 17 subclasses was added to each sample (AB Sciex, Redwood City, CA, USA 5040156, Avanti, Alabaster, AL, USA 330827, Avanti 330830, Avanti 330828, and Avanti 791642) (Supplementary Table S1) prior to biphasic extraction. After two successive extractions, pooled organic layers were dried in a Thermo, Waltham, MA, USA, SpeedVac SPD300DDA (ramp setting 4 at 35 °C for 45 min, total run time of 90 min). The samples were resuspended in 1:1 methanol/dichloromethane with 10 mM ammonium acetate and transferred to robovials (Thermo 10800107) for analysis.

Analysis of the samples was undertaken on the Sciex 5500/DMS device (Lipidyzer Platform, SCIEX, Framingham, MA, USA) with an expanded targeted acquisition list consisting of 1450 lipid species across 17 subclasses. The Differential Mobility Device on Lipidyzer was tuned with EquiSPLASH LIPIDOMIX (Avanti 330731). For the tuning process, the MRMs for the single class standards in the EquiSPLASH mix were acquired while the Compensation Voltage (CoV) was ramped to determine optimal DMS settings for separation and sensitivity for each lipid subclass. The optimal CoV settings were then applied to all measured species within the DMS shotgun lipidomics method. The 1450 targeted MRMs in the assay were acquired during two infusions (one using the DMS and the second without the DMS) with MRMs acquired in both negative and positive mode (Supplementary Material Table S2A–D). In-house data analysis platform (DMS Shotgun Lipidomics Assistant (SLA), Python software is an open-source application) is comparable to the Lipidyzer Workflow Manager [36] was used. Further details on the instrument method, settings, and data analysis are described in a previous publication [36] and the associated Github entry. Quantitative plasma and milk values were normalized to volume (mls), and concentrations are reported as nmoles/mL.

*Statistical Analysis:* Maternal plasma ω3 and ω6 PUFA data were compared between OW/OB and normal BMI mothers using an unpaired t-test. Milk ω3 and ω6 PUFA composition between the sample aliquot (first and last) and maternal BMI was analyzed by repeated measures of ANOVA. Linear regression was used to test the correlation between maternal plasma and milk FAs with maternal BMI. Normality was checked using the Shapiro–Wilk test. All analyses included *n* = 9 Normal BMI and *n* = 11 OW/OB, with values expressed as means ± SEM.

All analyses were performed using GraphPad Prism (Version 10.0.0, GraphPad Software Inc., San Diego, CA, USA), and statistical significance was set at *p* < 0.05.

## 3. Results

### 3.1. Profile of Study Women

A detailed description of the characteristics of the studied women, including breast milk composition, has been previously reported [31], with pertinent data provided in Table 1. Briefly, 22 women (5 Asian, 12 Hispanic, 4 Black, and 1 White non-Hispanic) were studied, of which 13 were OW/OB (BMI 32.5 ± 1.5) and 9 were normal (BMI 21.2 ± 0.6). Among the serum values, women with OW/OB had significantly increased insulin (17.6 ± 3.4 vs. 8.4 ± 2.0 µIU/mL, *p* = 0.04), though with comparable triglyceride levels. Notably, these women produced milk with consistently higher fat (*p* = 0.01) and caloric (*p* = 0.005) content as evident in fore- and hind milk aliquots—(First milk sample: Fat, 2.2 ± 0.4 vs. 1.3 ± 0.2 mg/dL; Calorie, 60 ± 3 vs. 53 ± 2 kcal and Last milk sample: Fat, 7.6 ± 0.8 vs. 5.8 ± 0.7 mg/dL; Calorie, 109 ± 7 vs. 92 ± 6 kcal). Based on these differences in fat content, the current study undertook a detailed analysis of PUFA.

### 3.2. Reduced Maternal Plasma ω3 and ω6 Fatty Acids in Women with OW/OB

**Figure 1:**
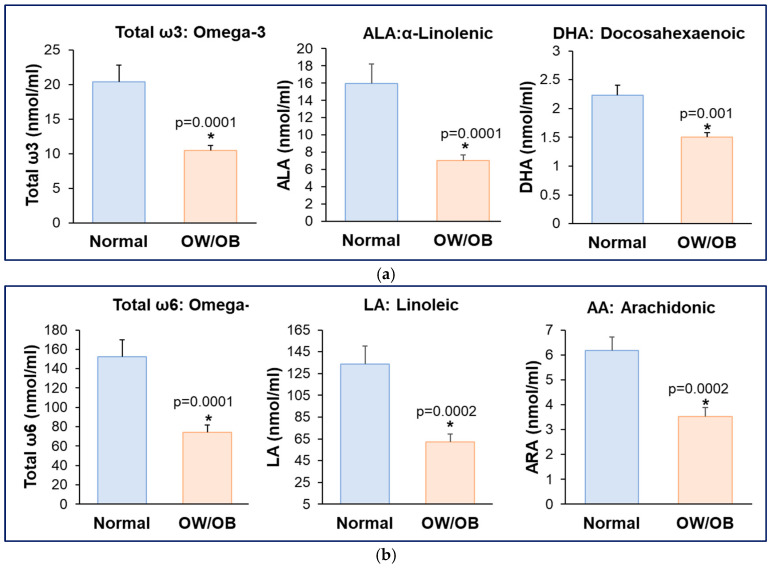
**Reduced maternal plasma ω3 and ω6 fatty acids in women with OW/OB.** (**a**) Maternal plasma ω3 PUFA (ALA, DHA) levels; (**b**) Maternal plasma ω6 PUFA (LA, AA) levels. Values are mean ± SEM of Normal BMI (*n* = 9) and OW/OB (*n* = 11). * OW/OB vs. Normal BMI.

In women with OW/OB, plasma total ω3 fatty acids, including α-linolenic acid (ALA) and docosahexaenoic acid (DHA) levels were significantly reduced (Figure 1a) as were the levels of plasma total ω6, linoleic (LA), and arachidonic (AA) acids (Figure 1b).

**Figure 2:**
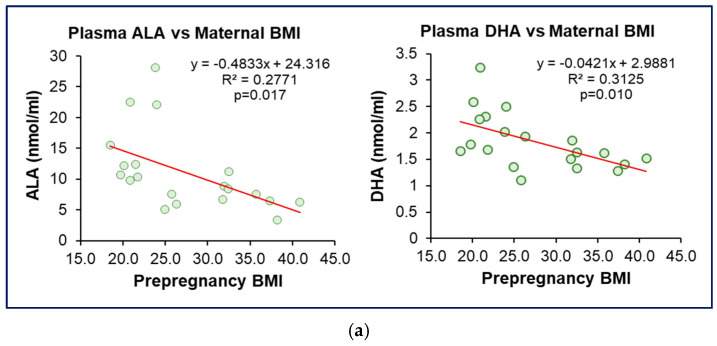

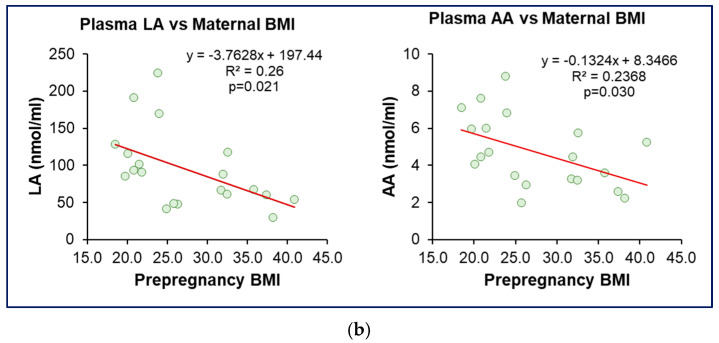
**Effect of Maternal BMI on Plasma Omega Fatty Acids.** (**a**) Linear regression of maternal plasma ω3 PUFA (ALA, DHA) vs. maternal pre-pregnancy BMI. (**b**) Linear regression of maternal plasma ω6 PUFA (LA, AA) vs. maternal pre-pregnancy BMI. *n* = 20 of the combined OW/OB and Normal BMI groups.

Consistent with this, plasma ω3 fatty acids (ALA, DHA) and ω6 fatty acids (LA, AA) levels showed a graded inverse correlation with maternal BMI (Figure 2a,b).

### 3.3. Increased Milk ω3 and ω6 Fatty Acids in Women with OW/OB

**Figure 3:**
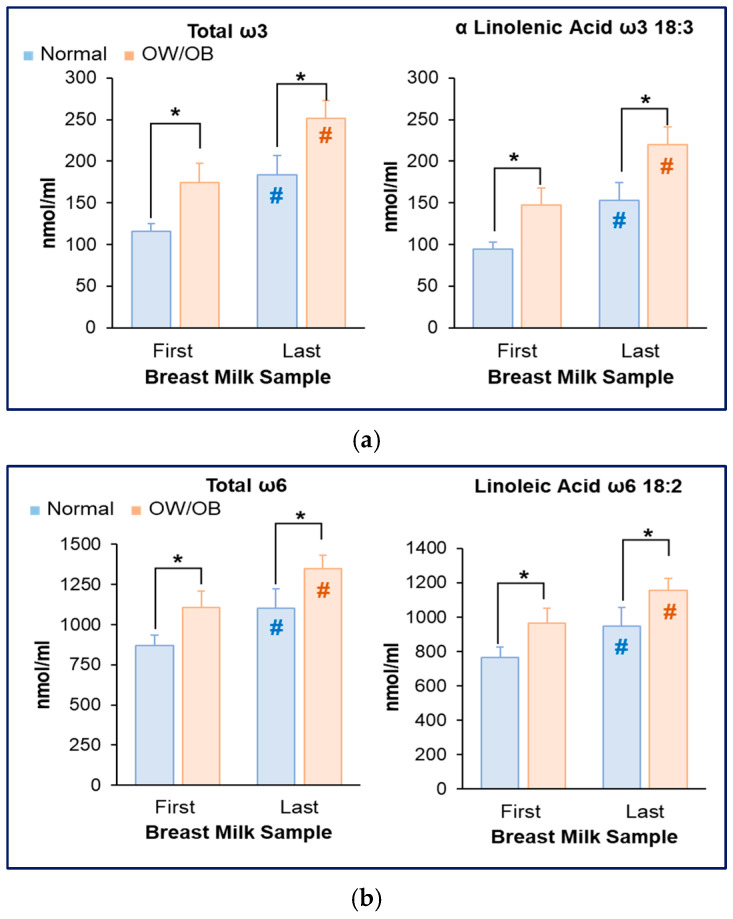
**Increased milk ω3 and ω6 in women with OW/OB.** (**a**) Breast milk content of total ω3 and ALA fatty acids in first and last milk sample aliquots. (**b**) Breast milk content of total ω6 and LA fatty acids in first and last milk sample aliquots. Normal BMI (*n* = 9) and OW/OB (*n* = 11). * *p* = 0.001 OW/OB vs. Normal; ^#^ *p* = 0.001 First vs. Last milk sample.

Consistent with the increased milk fat content, total ω3 and ω6 FAs, including ALA and LA (precursors of LCFAs), were significantly increased in first (foremilk) and last (hindmilk) milk samples of women with OW/OB. Overall, amongst all women, levels of ω3 and ω6 FAs increased from the first to the last milk sample (Figure 3a,b).

### 3.4. Reduced Milk DHA and Increased AA Content in Women with OW/OB

**Figure 4:**
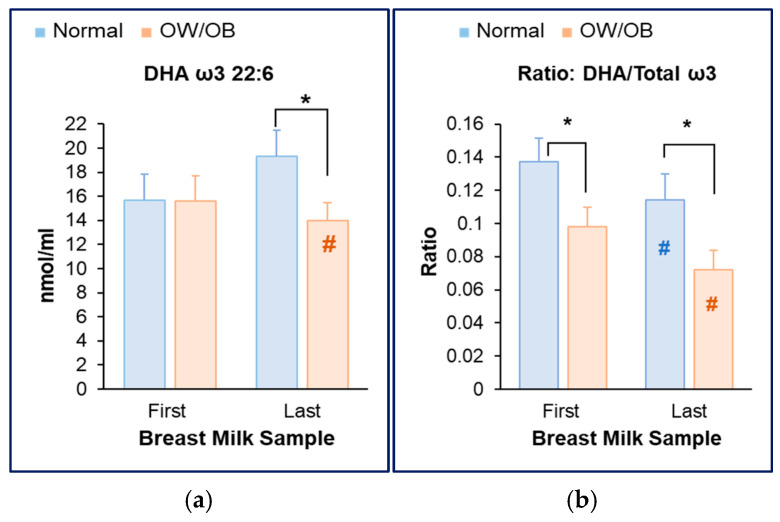
**Reduced Milk DHA content in women with OW/OB.** (**a**) Breast milk DHA content in the first and last milk sample aliquots. (**b**) Breast milk ratio of DHA to total ω3 in first and last milk sample aliquots. Normal BMI (*n* = 9) and OW/OB (*n* = 11). * *p* = 0.01 OW/OB vs. Normal; ^#^ *p* = 0.01 First vs. Last milk sample.

Despite higher breast milk fat content and higher total ω3 and ω6 FAs, DHA levels were significantly reduced (last milk sample) in OW/OB versus normal BMI subjects (Figure 4a). Thus, when expressed relative to total omega (ω3 and ω6) FAs, the DHA/Total ω3 ratio was significantly decreased in the first and last milk samples of women with OW/OB (Figure 4b).

### 3.5. Increased AA Content in Women with OW/OB

**Figure 5:**
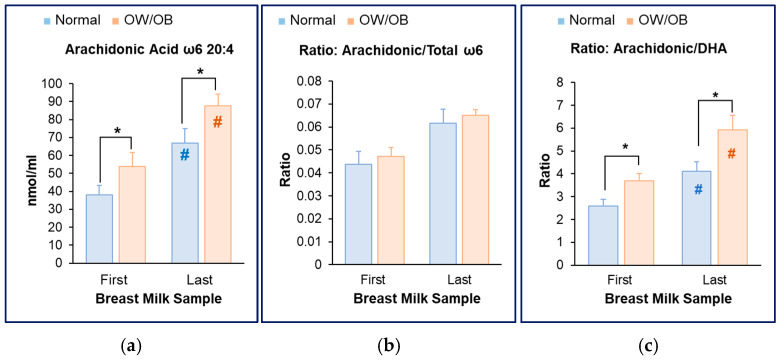
**Increased milk AA content in women with OW/OB.** (**a**) Breast milk AA content in first and last milk sample aliquots. (**b**) Breast milk ratio of AA to total ω6 in first and last milk sample aliquots. (**c**) Breast milk ratio of AA to DHA in first and last milk sample aliquots. Normal BMI (*n* = 9) and OW/OB (*n* = 11). * *p* = 0.01 OW/OB vs. Normal; ^#^ *p* = 0.01 First vs. Last milk sample.

In contrast to DHA content, breast milk AA levels were significantly increased in both the first and last milk samples of women with OW/OB as compared to normal weight (Figure 5a). Consistent with this, the ratio of AA/Total ω6 was comparable in both groups of women (Figure 5b). Importantly, the AA/DHA ratio was significantly increased in foremilk and hindmilk of women with OW/OB (Figure 5c).

### 3.6. Association Between Milk Content and Maternal Plasma Levels

**Figure 6:**
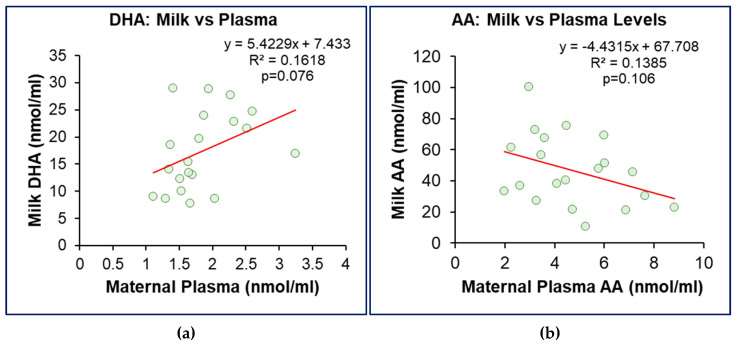
**Association between milk DHA and AA Content and maternal plasma levels.** (**a**) Linear regression of breast milk DHA vs. maternal plasma DHA levels. (**b**) Linear regression of breast milk AA vs. maternal plasma AA levels. *n* = 20 of the combined OW/OB and Normal BMI groups.

Whilst breast milk DHA levels showed a positive correlation with maternal plasma DHA levels (Figure 6a), breast milk AA demonstrated a negative correlation with maternal plasma AA (Figure 6b).

## 4. Discussion

To examine milk composition, we utilized a method of breast pumping which provides a cross-section of milk composition from foremilk to hindmilk, controlling for the number of weeks of postpartum nursing, time of day, and the interval from prior breast emptying [31]. Utilizing samples of 10 mL, we previously demonstrated a four-fold increase in milk fat content from the first foremilk to the last hindmilk sample [31]. In the present study, we demonstrated marked differences in maternal plasma and breast milk ω3 and ω6 FA concentrations among women with OW/OB, indicating significant differences in nutritional exposures for their infants.

Plasma levels of both ω3 and ω6 PUFA levels were significantly lower in OW/OB subjects, with total ω3 and ω6 FA levels 50% in women with normal BMI. Similarly, plasma ALA, DHA, LA, and AA demonstrated significant inverse correlations with maternal pre-pregnancy BMI. These associations likely represent dietary as well as potential metabolic differences. In adult women, the average daily ALA intake is 1.59 g [37], whereas DHA intake is less than 100 mg [38]. Hepatic conversion of both ω6 LA and ω3 ALA to longer chain ω6 and ω3 FAs, respectively, occurs via parallel pathways of Δ-5 and Δ-6 desaturases and elongase [39], which increase activity in response to insulin [40]. Due to the dual pathway interaction, maternal diets high in ω6 reduce ω3 PUFAs, as a result of inhibition of Δ-5 and Δ-6 desaturase [41]. The worldwide dietary changes associated with a Western diet have led to a marked increase in ω6 plant oils and thus a decrease in both DHA dietary consumption and the metabolic conversion of ALA to DHA [42,43]. Higher insulin levels in OW/OB women (17.6 ± 3.4 vs. 8.4 ± 2.0 µIU/mL) [31], as previously reported, may impact these pathways.

Consistent with the increased levels of total fat content in breast milk of OW/OB women, total ω3 and ALA and total ω6, LA, and AA milk concentrations were significantly increased in both foremilk and hindmilk samples of OW/OB women. In contrast, milk DHA levels in OW/OB women were similar in foremilk and significantly decreased in hindmilk samples as compared to normal BMI women. Thus, the ratio of DHA/Total ω3 was significantly decreased in both foremilk and hindmilk samples of OW/OB women. The resulting increase in the ratio of AA/DHA in OW/OB women may potentiate a proinflammatory environment.

**Figure 7:**
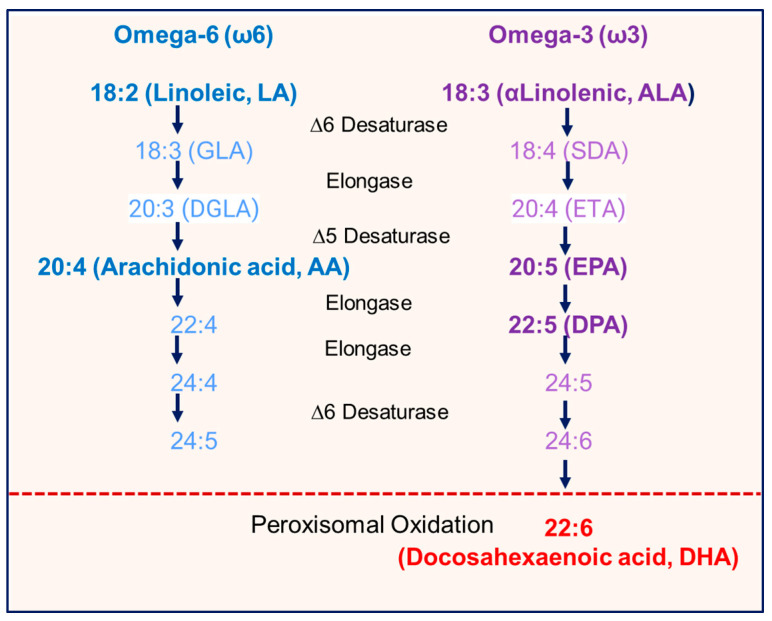
**Omega-6 (ω6) and Omega-3 (ω3) metabolic pathway**. 18:2n-6 linoleic (LA) and 18:3n-3 α-linolenic acid (ALA) are precursors of longer chain C20 and C22 PUFAs, including arachidonic (AA), eicosapentaenoic (EPA), and docosahexaenoic acids (DHA). DHA requires peroxisomal oxidation, which is a rate-limiting step in the conversion of ALA to DHA.

Importantly, infants are dependent upon milk DHA intake, as the majority of infants cannot synthesize enough DHA from milk ALA [44]. Similarly to hepatic processes, mammary epithelial cells convert LA and ALA to longer chain PUFAs. In contrast to ω6 eicosanoids and ω3 EPA and DPA, DHA specifically requires peroxisomal oxidation, a rate-limiting step in ALA to DHA conversion (Figure 7). There is limited understanding of mammary peroxisome function as it relates to milk essential fatty acid content. High-fat diet, obesity, insulin, and reactive oxygen species suppress peroxisomal activity [45,46], consistent with our findings of reduced ratios of DHA/Total ω3 ratio in milk of subjects with OW/OB. These findings may explain the 10-fold individual variability in the mammary conversion of ALA to DHA (0.5 to 5%) [47].

The increased milk AA to DHA ratio may potentiate a proinflammatory intestinal exposure [48,49,50] and potentially impact infant gastrointestinal function and the risk of necrotizing enterocolitis [51]. Absorption of milk PUFAs may have long-term neurological and developmental effects. Studies consistently suggest better health outcomes for preterm and term infants consuming milk with higher concentrations of ω3 (ALA, EPA, and DHA) and negative health outcomes associated with higher ω6 (LA and AA) [52,53,54]. Higher milk ω3 PUFAs were associated with improved infant affectivity at 6 months [55], while infant cognition at 18 months was negatively affected by reduced milk ω3:ω6 ratio [56]. Among children (5–12 years), low plasma DHA was associated with attention deficit/hyperactivity disorder [57]. Maternal supplementation with ω3 as compared to ω6 PUFAs during pregnancy and lactation was demonstrated to increase early childhood IQ scores in one [58] though not all studies [59], whereas other studies have demonstrated DHA-improved sequential processing [42,43] and eye–hand coordination [60]. A generational effect from mothers to offspring may be apparent as a lower ratio of cord plasma ω3:ω6 PUFAs was associated with higher odds of childhood obesity [61].

The finding that breast milk DHA correlates positively with maternal plasma DHA supports the concept of maternal DHA supplementation during late pregnancy or postpartum. The negative correlation of breast milk AA with maternal plasma AA indicates that OW/OB subjects (with lower maternal plasma AA concentration) preferentially transfer AA into breast milk. As AA is essential, but potentially inflammatory, this imbalance of ω3 and ω6 FAs suggests that approaches to limit plasma to breast milk AA transfer in OW/OB subjects may be of value.

Despite the power calculation, further research in this area with a larger sample size and a longer fasting period for maternal blood collection would strengthen the data.

## 5. Conclusions

In summary, women with OW/OB produce breast milk with increased total fat and caloric content [31], though reduced milk DHA. We hypothesize that these findings likely represent, in part, a consequence of reduced mammary peroxisomal conversion of ALA to DHA, resulting from insulin or reactive species within the maternal obese environment [42,43,51,52,53,54,55,56,57,58,59,60,61]. Strikingly, worldwide DHA intake during pregnancy is far below recommendations, and the average US intake is less than 30% of the recommended [25,38,62]. Although maternal DHA supplementation increases maternal plasma and milk DHA levels and increases infant plasma ω3:ω6 ratio [39,63], DHA supplementation is not widely used. These findings support the consideration of both maternal and perhaps infant DHA supplementation throughout pregnancy and nursing. In parallel to nutritional approaches, an enhanced understanding of mammary uptake, enzymatic conversion, processing, and secretion of essential PUFAs may provide therapeutic approaches to normalize breast milk DHA production in OW/OB subjects.

## Figures and Tables

**Table 1:** Maternal demographics.

	Normal (BMI 18.9–24.9) *n* = 9	OW/OB (BMI ≥ 25) *n* = 13	*p*-Value OW/OB vs. Normal
Age	29.8 ± 2.6	29 ± 1.9	NS
Pre-pregnancy Height (m)	1.60 ± 0.02	1.59 ± 0.01	NS
Pregnancy Weight (kg)	54.2 ± 1.5	81.9 ± 3.3	*p* < 0.001
Pre-pregnancy BMI (kg/m^2^)	21.2 ± 0.6	32.5 ± 1.5	*p* < 0.001
Gestational Weight Gain (kg)	14.8 ± 1.8	11.8 ± 1.1	*p* = 0.08

Values are Mean ± SEM. NS = not significant.

## Data Availability

The data presented in this study are available from the corresponding author upon reasonable request. The data are not publicly available due to privacy or ethical restrictions.

## Supplementary Materials

The following supporting information can be downloaded at: https://www.mdpi.com/article/10.3390/nu17132158/s1, Table S1: Standard; Table S2: 2A Method 1 Neg Mode, 2B Method 1 Pos Mode, 2C Method 2 Neg Mode, 2D Method 2 Pos Mode.

## Abbreviations

The following abbreviations are used in this manuscript:

OW/OB	Overweight/obese
FA	Fatty Acid
PUFAs	Polyunsaturated Fatty Acids
LA	18:2n-6 linoleic
ALA	18:3n-3 α-linolenic acid
AA	Arachidonic Acid
DHA	Docosahexaenoic Acid
LCFA	Long-chain fatty acid
ω3	Omega-3 fatty acids
ω6	Omega 6-fatty acids
BMI	Body mass index
